# Narrow QRS Complexes in a Patient with Baseline Surgical RBBB: What is the Mechanism?

**Published:** 2010-08-10

**Authors:** Harinder R Singh, Srinath Gowdagere

**Affiliations:** Division of Cardiology, The Carman and Ann Adams Department of Pediatrics, Children's Hospital of Michigan, Wayne State University School of Medicine, Detroit, MI 48201

**Keywords:** Tetralogy of Fallot, surgical RBBB, PVC

## Case Presentation

**Figure 1:**
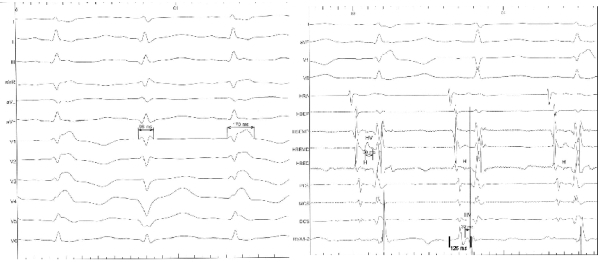
Right shows a 12 lead electrocardiogram showing spontaneous narrow complex QRS interspersed between wide QRS complexes with complete RBBB morphology. Left shows simultaneously obtained intra-cardiac tracings with no change in PR and AH intervals and slightly shortened HV interval (32 msec as compared to 50 msec with the normal beats) with evidence of a premature ventricular activation in the RV apical catheter.

A 26-year-old woman born with Tetralogy of Fallot initially underwent a left Blalock-Taussig shunt followed by a complete repair with a transannular patch at 2 years of age. She complained of non-sustained short episodes of palpitations. Her Holter monitor showed episodes of six beat run of non-sustained ventricular tachycardia. She was scheduled to undergo surgical pulmonary valve replacement. In view of her symptoms and Holter results she underwent an electrophysiology study to map any substrate of ventricular tachycardia for intra-operative ablation. Her baseline electrocardiogram demonstrated complete right bundle branch block pattern (RBBB) with QRS duration of 174 msec secondary to surgical interruption of the right bundle branch. During the electrophysiology study she was noted to have intermittent narrow QRS complexes (Figure 1).The QRS complexes had a duration of 98 msec with no change in the PR interval and AH interval. What is the mechanism of narrow QRS complexes in this patient with baseline right bundle branch block (RBBB) pattern?

## Commentary

On initial assessment of the intracardiac tracings as well as the 12 lead electrocardiogram, there is evidence of baseline RBBB (surgical) with intermittent appearance of normally conducted sinus activations with normal AH and slightly shortened HV. However on further analysis there seems to be a premature ventricular complex from the right ventricular apex that occurs immediately after the His deflection causing the apparent narrowing of the QRS complexes.

Intermittent transition from wide to narrow QRS complex has been described with multiple mechanisms. These include change in heart rate, 'peel-back' refractoriness, rate dependent progressive shortening of bundle branch refractoriness, gap phenomena, supernormality, loss of pre-excitation, premature ventricular complex (PVC) ipsilateral to the bundle branch block and equal conduction delay in both the bundle branches [1]. Presence of a surgical RBBB rules out the possibility of either change in heart rate, 'peel-back' refractoriness, or rate dependent progressive shortening of bundle branch refractoriness as a mechanism for transition to narrow QRS complex. Gap phenomenon is an explanation for failure of a premature atrial impulse to conduct but resumption of conduction with even earlier premature extrastimuli. It occurs when the functional refractory period of tissue proximal in the conducting system is shorter than the effective refractory period of distal conducting tissue [2]. In our patient we see no evidence of premature atrial complex and no change in conduction intervals. Supernormality is better conducitivity and excitability during phase IV at the period of dip in membrane electrical resistance. This is seen usually at the terminal end of T waves or during the U waves [3]. This is not evident in our case. Equal conduction delay in both the bundle branches can lead to narrow QRS complexes but in our case there is no delay in the left bundle evident from the intracardiac tracings. Premature ventricular complex ipsilateral to bundle branch block can cause normal QRS duration.  The sinus beat is normally conducted and travels to the left and at the same time the premature ventricular complex from the right ventricle depolarizes the right ventricle simultaneously causing a narrow QRS complex and a shortened HV interval. This proposed explanation is then further validated by reproducing a narrow QRS complex by a properly timed premature ventricular stimulation (based on the timing of the spontaneous PVC) at the right ventricular apex (Figure 1). The timed premature ventricular stimulation produced exactly identical narrow QRS complex with the same intervals and morphology. The spontaneous PVC could be catheter induced; however, the physiologic principle remains the same. This case illustrates the physiology that has been described before, in a patient with a surgical RBBB who spontaneously was detected to have normal QRS complexes. We unequivocally reproduced normalization of QRS duration with timed PVC ipsilateral to the bundle branch block.

This physiological mechanism may have potential clinical applications for optimizing the timing of pacing intervals to obtain narrow QRS complexes as well to obtain interventricular synchrony.

## Figures and Tables

**Figure 2:**
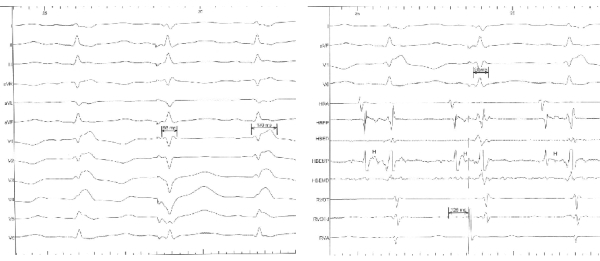
Right shows a 12 lead electrocardiogram showing a programmed premature ventricular stimulus producing an identical narrow QRS complex as seen in Figure 1. Left shows simultaneously obtained intra-cardiac tracings with comparable PR, AH and HV intervals and the timing of S2 (126 msecs after the high atrial activity) comparable to the spontaneous PVC evident in Figure 1.
